# Finite velocity of ECG signal propagation: preliminary theory, results of a pilot experiment and consequences for medical diagnosis

**DOI:** 10.1038/s41598-023-29904-2

**Published:** 2023-03-22

**Authors:** Teodor Buchner, Maryla Zajdel, Kazimierz Pȩczalski, Paweł Nowak

**Affiliations:** 1 Faculty of Physics, Warsaw University of Technology, Warsaw, Poland; 2 Faculty of Mechatronics, Warsaw University of Technology, Warsaw, Poland

**Keywords:** Biophysics, Cardiology, Applied physics, Biological physics

## Abstract

A satisfactory model of the biopotentials propagating through the human body is essential for medical diagnostics, particularly for cardiovascular diseases. In our study, we develop the theory, that the propagation of biopotential of cardiac origin (ECG signal) may be treated as the propagation of low-frequency endogenous electromagnetic wave through the human body. We show that within this approach, the velocity of the ECG signal can be theoretically estimated, like for any other wave and physical medium, from the refraction index of the tissue in an appropriate frequency range. We confirm the theoretical predictions by the comparison with a direct measurement of the ECG signal propagation velocity and obtain mean velocity as low as v=1500 m/s. The results shed new light on our understanding of biopotential propagation through living tissue. This propagation depends on the frequency band of the signal and the transmittance of the tissue. This finding may improve the interpretation of the electric measurements, such as ECG and EEG when the frequency dependence of conductance and the phase shift introduced by the tissue is considered. We have shown, that the ECG propagation modifies the amplitude and phase of signal to a considerable extent. It may also improve the convergence of inverse problem in electrocardiographic imaging.

## Introduction

Albert Einstein was right that “It is the theory, which decides what we can observe.” On rare occasions in physics, we can perform relatively simple, even trivial experiments, which open new horizons for sometimes quite remote branches of science and technology if equipped with novel theoretic explanations.

Specifically, this is the case for biopotentials, which are used worldwide in the context of electrocardiography (ECG) or electroencephalography (EEG) and numerous other techniques. We asked ourselves a fundamental question: how fast is the ECG signal? The question opened a particularly interesting and poorly explored research subject. Many theories for ECG propagation have been formulated so far^1,2^. They generally assume, that the ECG propagates at the speed of light. The galvanic (conductive) coupling between the source and the neighboring tissue is based on microscopic Ohm law, which assumes, that the response to the altered potential of the source appears instantly in the studied volume, such as the torso. This low level assumption formulates a starting point for modelling of biopotential propagation in the tissue, which is shared by many other methods, and makes a difference in how the results are interpreted and measurements are optimized.

Such research techniques as electrical impedance tomography (EIT) have a potentially high impact in many areas: diagnosis of ischemic brain injury^3^, brain hypoxia^4^, epilepsy and cerebral edema^5^, congestive heart failure^6^ hypertension^7^, and importantly in wide area of oncology^8^. The authors underline a technical simplicity, compared to other methods, which allows using the EIT even in battlefield applications^5^, when monitoring and triaging trauma patients is of paramount importance^9^. However, despite a particularly rich area of application, which extends far from physiological applications - to industrial contexts^10^, or even military applications^11^, the performance of EIT techniques is far from perfect - as expressed e.g. by Sergi et al.^12^. Is it possible, that one of the reasons may be the wrong assumption concerning the basic model? The same applies to techniques of Electrocardiographic Imaging (ECGI)^13^, which despite vivid development, have not managed to enter the guidelines for the management of cardiological patients^14,15^.

In these two techniques, a question of paramount importance, but relatively rarely addressed, is the measurement model, which is generally considered to be entirely based on tissue conductance^1,2,16,17^. We are not addressing the validity of this model in general; we only want to show that a rich physical context for the basic biomedical measurements opens up if we base our model on the dielectric permittivity of the tissue. Our preliminary findings, showing a particularly rich physical background in tissue modelling^18^ follows the footsteps of other experimental and theoretical efforts^19–21^ and modelling studies^8,22^, to name just a few. As far as we know, the velocity of the ECG signal has never been measured, apart from our own early finding that at 1 kHz sampling the effects of the finite velocity cannot be observed^23^.

It is commonly known that the source of the ECG signal is the cyclic transport of charges across the cell membrane of cardiomyocytes^24^. Typically the interest in the extracellular charge ends at this statement, because in further considerations only a somewhat less intuitive term biopotential is used, obscuring the actual physical reality^1^. We assume, that the physics of the ECG is based on transport of charge, which modifies the spatial distribution of charge in the extracellular space. Depending on the direction of the net membrane current, this excess charge loads or unloads the generally non-conducting membranes, such as epicardium, which are entirely formed of connective tissue. There seems to be no physical transport of charge through the epicardium and the pericardial sac, which may be directly deduced from the fact, that the composition of electrolytes in pericardial fluid is different to that of extracellular fluid and also to that of the blood plasma^25^. The actual anatomy of the epicardium is never considered in the literature. We further assume, that the charge appearing at the epicardium affects the state of thermodynamic equilibrium between the heart and the surrounding tissues, even though the epicardium can be considered as a source of EM wave (like a plate of a capacitor), according to fundamental laws of electromagnetism^26^. In our paper we show the preliminary semi-empirical estimation of propagation velocity using a simplified model. The formula describing the propagation velocity of EM wave through a physical medium can be derived from Maxwell’s equations. For linear, isotropic, dispersive medium, propagation velocity depends on the frequency of the source of the EM wave and the refractive index of the considered medium of propagation^26^, as expressed in Eq. (1):

1 $$\begin{aligned} v(f) = \dfrac{c}{n(f)}. \end{aligned}$$

Here:

- *v* - propagation velocity of EM wave through a physical medium
- *n* - refractive index of the physical medium
- *f* - EM wave frequency
- $c=3\times 10^8$ m/s - speed of light in the vacuum

Analysis of the power spectrum of ECG signal reveals that the largest relative power is carried by frequencies in the range around 2–10 Hz^1,27^. Therefore, this frequency range must be considered the most significant component in estimating the value of ECG signal propagation velocity through the human body.

The refractive index depends on the electric permittivity $\varepsilon _r$ and magnetic permeability $\mu _r$ of the medium, as shown in Eq. (2). For simplification, we consider that our medium is composed only of muscle tissue, which is motivated by our experimental setup. Magnetic permeability $\mu _r$ of muscle tissue can be neglected as this value is almost equal to one^1^, which makes it several orders of magnitude smaller than the electric permeability, as we show below.

2 $$\begin{aligned} n = \sqrt{\varepsilon _r \mu _{r}}. \end{aligned}$$

Here:

- *n* - refractive index of the physical medium
- $\varepsilon _r$ - electric permittivity of the medium
- $$\mu _r$$ - magnetic permeability

Therefore the final formula, shown in Eq. (3) is particularly simple:

3 $$\begin{aligned} v(f) = \dfrac{c}{\sqrt{\varepsilon _r(f)}} \end{aligned}$$

Here:

- *v* - propagation velocity of EM wave through a physical medium
- *f* - EM wave frequency
- $c=3\times 10^8$ m/s - speed of light in the vacuum
- $\varepsilon _r$ - electric permittivity of the medium

In the current literature, there is practically no data on the relative electrical permeability of biological tissues below 10 Hz since this area was not considered important. To estimate the value of propagation velocity for the considered medium, we refer to the fundamental work of Gabriel et al., where electrical permittivity of many tissues in frequency range: 10–20 GHz is measured^28^. For muscle tissue at $f=10$Hz Gabriel et al. report $\varepsilon _r=10^8$—a value rather rarely found in optical papers. At such a low frequency, multiple mechanisms for tissue polarisation have their critical time shorter than the period of the EM field. It follows from 3, that the approximate propagation velocity of 10 Hz EM wave through muscle is 30,000 m/s. Propagation velocity of ECG signal, consisting of a rich spectrum of frequencies, through multiple layers of different dispersive biological tissues can’t be given as one value since each component frequency propagates with different velocity through particular types of tissues. Therefore, the above calculations are an estimate of the expected order of magnitude. Considering that the electrical permittivity $\varepsilon _r$ of the body tissue is a decreasing function of the frequency, by extrapolation of currently available data, we can expect that the $\varepsilon _r$ at $f<10$ Hz is going to be even higher. Therefore the above approximation defines an upper limit for the measured value.

The objective of this work is to validate this theoretical result on endogenous biopotential propagation velocity. The experimental setup for this validation is described below.

## Related work

The electric activity of the heart was first reported by Waller as early as 1889^29^, but due to the Nobel-awarded research of Einthoven^30^, followed i.a. by Goldberger and Wilson, it has achieved its modern shape^31^. The history of clinical expansion of cardiac biopotentials is well known and easy to track. It has led to many spectacular results, such as identification of various specific types of arrhythmia: atrial fibrillation^32^, ventricular ectopy^15^ or supraventricular tachycardia^33^, identification of changes related to myocardial infarction^34^ atrioventricular blocks, long QT syndrome^35^. The measurable symptoms of numerous other aberrations of rhythm and conductance were also identified and reported in clinical sciences. It is also easy to track the progress in measuring equipment^1,16^. Since Einthoven or Holter who had deep understanding of the measuring equipment, the clinicians used to constantly challenge the bioengineers, by demanding the equipment, which would be more precise, miniaturized or robust to noise.

Substantially less attention was attracted to the phenomenon of actual conductance of cardiac biopotentials. Although their microscopic origin is well known^1,36^, the actual propagation was virtually never studied on the clinical level. One of the rare findings was provided by the analysis of proximal placement of the electrodes - the so-called Mason-Likar 12-lead system^37^. It was found that some changes in the ECG, result from the altered positions of the electrodes^38^. New electrode setups have been proposed to overcome this problem^39^, but to our knowledge no biophysical analysis of this phenomenon was performed at that time. Earlier, Katz, Sigman and Kaufman and their coworkers have analyzed various effects related to the vicinity of the heart, including an early version of the theory of heart and the phenomenon of shunting^40–42^. Geselowitz developed a theory of the ECG based on his idea of impressed current^2^, which was further developed and described in depth by Malmivuo and Plonsey^1^. The electric properties of the tissue have been studied at various occasions, starting from Höber, who suggested, that the essential property of the living tissue is its conductance^43^. Interestingly, after Geselowitz, the phenomenon of the ECG was never analyzed in context of complex transmittance of the tissue, although the transmittance of the Ag/AgCl electrodes was studied often^44^. The human body was treated as a composition of tissue patches, labelled by conductances, which were treated as scalars or matrices (in case of anisotropy)^1^. Such an approach leaves apart the phenomenon of dispersion, i.e. the dependence of tissue properties on frequency, which was frequently studied in the context of RF techniques^28^, for patient or workmen health and safety. Due to this fact, as far as we know, our current work has no direct prior research, which we could cite directly.

## Methodology

We designed a specific electrode setup that enables measuring the ECG signal propagation velocity through the left upper limb. We noticed that while passing through the arm, the signal propagates through the medium of approximately cylindrical shape and relatively uniform tissue composition which simplifies the analysis of the measurements. All procedures performed in studies involving human participants were in accordance with the ethical standards of the institutional and/or national research committee and with the 1964 Helsinki Declaration and its later amendments or comparable ethical standards. All experimental protocols were approved by the ethical committee of Warsaw University of Technology. The measurement was performed on 3 healthy volunteers, members of the research team, who gave their informed consent, further denoted as #1, #2 and #3, respectively, 50 times for each subject.

**Figure 1:**
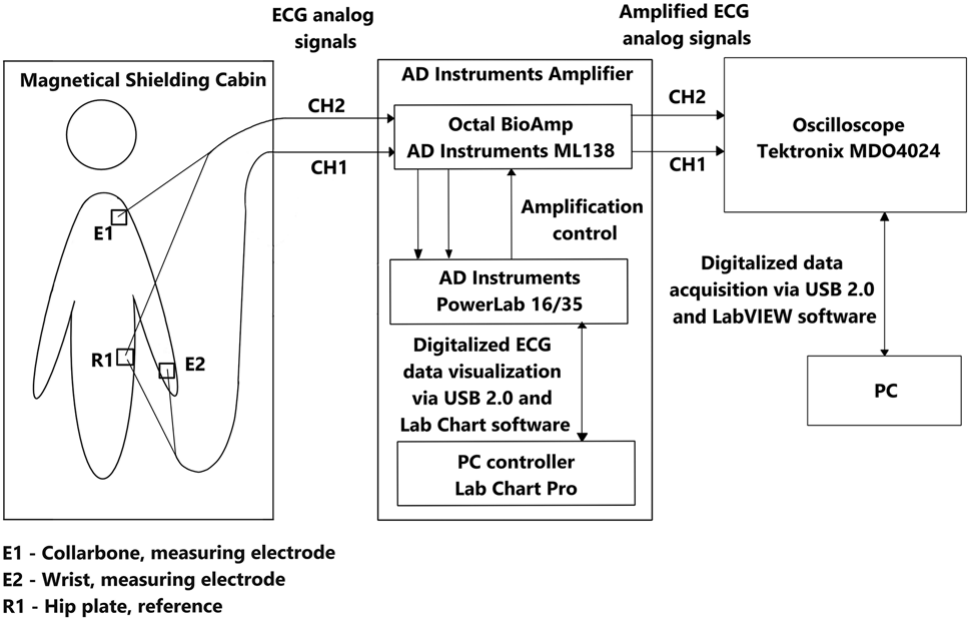
Measuring system for ECG signal propagation velocity.

The subject under test was placed in Magnetical Shielding Cabin (MSC) (VACOSHIELD 130, Vacuumschmelze GmbH & Co. KG, Hanau, Germany)) which isolated the patient from the surrounding EM field generated by the electrical network. The subject was sitting on a wooden chair, both hands resting on the armrests. ECG electrodes (Ambu BlueSensor R Ref. R-00-S/25) were used. The experimental setup is shown in Fig. 1. Measuring electrodes were placed on the left collar bone (E1) and on the left wrist (E2)

The distance *d* between measuring electrodes was measured, and the details are given in Table 1. The potential in measuring electrodes E1 and E2 was measured against the reference electrode R1 placed on the left hip plate, forming two nonstandard ECG leads: CH1, CH2 (Fig. 1).

**Table 1:** Study group characteristics.

Subject	Distance between measuring electrodes *d* [meters]	Age [years]
#1	0.55(0.05)	23
#2	0.64(0.05)	65
#3	0.63(0.05)	51

Both leads, CH1 and CH2, were passed by shielded cables to the AD Instruments Amplifier, which consists of an Octal Bio Amp (AD Instruments Octal Bio Amp Model ML138 Serial ML138-0299)^45^, data acquisition hardware (AD Instruments PowerLab 16/35 N12128)^46^ and a PC controller with the dedicated software (Lab Chart Pro v8.1.11)^47^ with auxiliary screen for primary visualization of the signal and parameters adjustment. In the PC controller, the following parameters were set for both channels: Range: 5 mV, Low pass: 200 Hz, High pass: 0.1 Hz, notch filter 50 Hz was applied. Note, that the measuring equipment was located outside the Magnetical Shielding Cabin.

Directly from AD Instruments Amplifier, both channels were fed to the oscilloscope (Tektronix MDO4024C, Tektronix Inc., USA)^48^. The signal was triggered from the less noisy channel - CH2. The steepest slope within QRS (usually the descending slope of the Q wave) was used for triggering. The sampling frequency of the oscilloscope was set to $f_s=5\,\text {MHz}$ so that three ECG cycles were visible on the screen. Afterward, the data from the oscilloscope were sent to the battery-powered PC, acquired by the software developed in LabView (National Instruments, LabView v.21.0, 64-bit) and further analyzed numerically in Python using a custom script. The source code of LabView and Python scripts along with the experimental data are available in the GitHub repository, http://github.com/teosbpl/HowFastIsTheECG, available from the corresponding author on reasonable request.

The most important parameter of measuring setup for proper analysis of the obtained signal is the sampling frequency, which equals 5 MHz - approximately 1000 times higher than in standard ECG devices. This high sampling frequency enables to observe the lag between two channels, which is the key parameter to compute the propagation velocity of the electromagnetic wave of cardiac origin. Another important parameter is the high signal to noise ratio to reliably determine the R peak position. Given the size of the pilot study group, the results of plain cross-correlation were sufficient, as they could be easily verified by visual inspection. However, many methods for R peak detection have been used since the classical paper by Pan and Tompkins^49^. The different peak and feature detection techniques are helpful in many specific applications, such as wearable devices^50,51^, arrhythmia detection^52,53^, or other, as summarized e.g. by Gupta et al.^54^. Finally, for the correlation to succeed, the QRS complex morphology has to be stable between channels, which we achieved by careful selection of electrode placement.

The signal to noise ratio in our measurements equals 12.45 dB, but the R peak is clearly visible.

We calculated the cross-correlation function using the Fast Fourier Transform mode of Scipy numerical library (v. 1.10)^55^. The computational complexity of this algorithm is $(3/2) N\log _2N - (3/2) N + 16$^56^. The time of particular cross-correlation computation is 1199.46 ms.

The cross-correlation function could be also computed directly from the defining formula (direct method), but the computational cost of this method was too high, since for the size of each channel of the order of $10^7$ samples each calculation took more than 10 minutes.

### Propagation velocity calculation

In order to measure propagation velocity, it is necessary to estimate the time lag between the waveforms in two channels of quite similar morphology. Of course, the morphology of the ECG changes while the signal passes through the tissue due to the transmission properties of the tissue. This may be formally represented by the tissue transmittance *H*(*f*). Here we leave these considerations for further research, and the problem of possible distortion will be neglected.

The number of lag samples $\Delta s$ between channels was computed from of the cross-correlation function of both channels. A typical waveform of the cross correlation of a trace containing three cycles is shown in Fig. 4a. The central part of the correlation plot, selected for further analysis, was limited to the order of magnitude predicted by the theory. The maximum value was determined using the Python function find_peaks.

The propagation velocity *v* was calculated as follows: sampling time $T_s$ was computed as a reciprocal of the sampling frequency, delay time *T* was calculated as $T = \Delta s T_s$, with a given sampling frequency $f_s$ of the oscilloscope and measured distance *d* between electrodes E1 and E2. The propagation velocity was calculated from the simple formula 4:

4 $$\begin{aligned} v = \dfrac{d}{T} \end{aligned}$$

Where:

- *v* - propagation velocity of EM wave through a physical medium [m/s]
- *d* - distance between measuring electrodes [m]
- *T* - delay time [s]

An example of the calculations for one measurement is summarized below. For $f_s=0.5\,\text {GHz}$ and measured lag $\Delta s=65006$ samples, with $d=0.55\,\text {m}$ we obtain $T_s=2\,\text {ns}$, $T=0.13\,\text {ms}$ and *v* calculated from Eq. (4) is $v=4231\,\text {m/s}$.

### Uncertainties of measurement

The measurement method presented above has the following sources of uncertainty: muscle noise, motion artifacts, skin-electrode contact potential, time-variant galvanic skin response, shielding cables inaccuracy, oscilloscope sampling frequency uncertainty and bio amplifier parameters, including the offset, the common mode rejection ratio and the finite input impedance. The distance between measuring electrodes is measured directly, and so is the maximum correlation. The formal approach includes calculating the uncertainty of ECG propagation velocity as the elementary propagation of uncertainty of direct measurements. We have found that the accuracy of these direct measurements introduces an error which is smaller by orders of magnitude than type A uncertainty related to the lag measurement, as described in the Results section, so further analysis in this direction was not performed.

### Ethical approval

All procedures performed in studies involving human participants were in accordance with the ethical standards of the institutional and/or national research committee and with the 1964 Helsinki Declaration and its later amendments or comparable ethical standards. All experimental protocols were approved by the ethical committee of Warsaw University of Technology (7/2022). Informed consent was obtained from all individual participants involved in the study.

## Results

**Figure 2:**
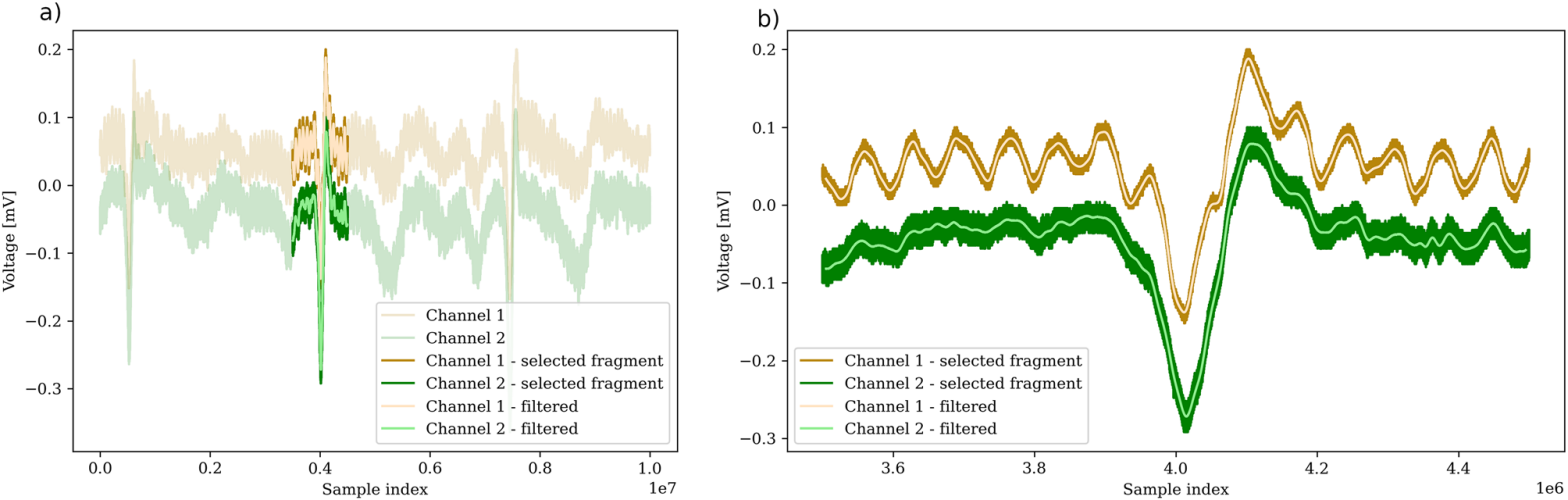
Example of the signal obtained for Subject #1. Figure (**a**) presents the wider scope of three cycles of ECG signal, Figure (**b**) presents the enlarged view of the particular QRS complex. Channel 1 is represented by a dark yellow color, Channel 2 is represented by a dark green color. Light yellow and light green represents signal filtered with a rolling mean filter with a rectangular window of size 5001 samples. Since the signal is obtained with an equipment of a sampling frequency approximately 1000 times higher than in standard ECG measurement, higher amplitude and frequency of noise is observed. However, the signal to noise ratio which equals 12.45 dB is satisfactory, as it allows to reliably determine the QRS complex position and cross-correlation can be computed. With lower sampling frequency signal to noise ratio increases (as can be seen in the (**a**) and (**b**), light yellow and light green color represents filtered signal).

**Figure 3:**
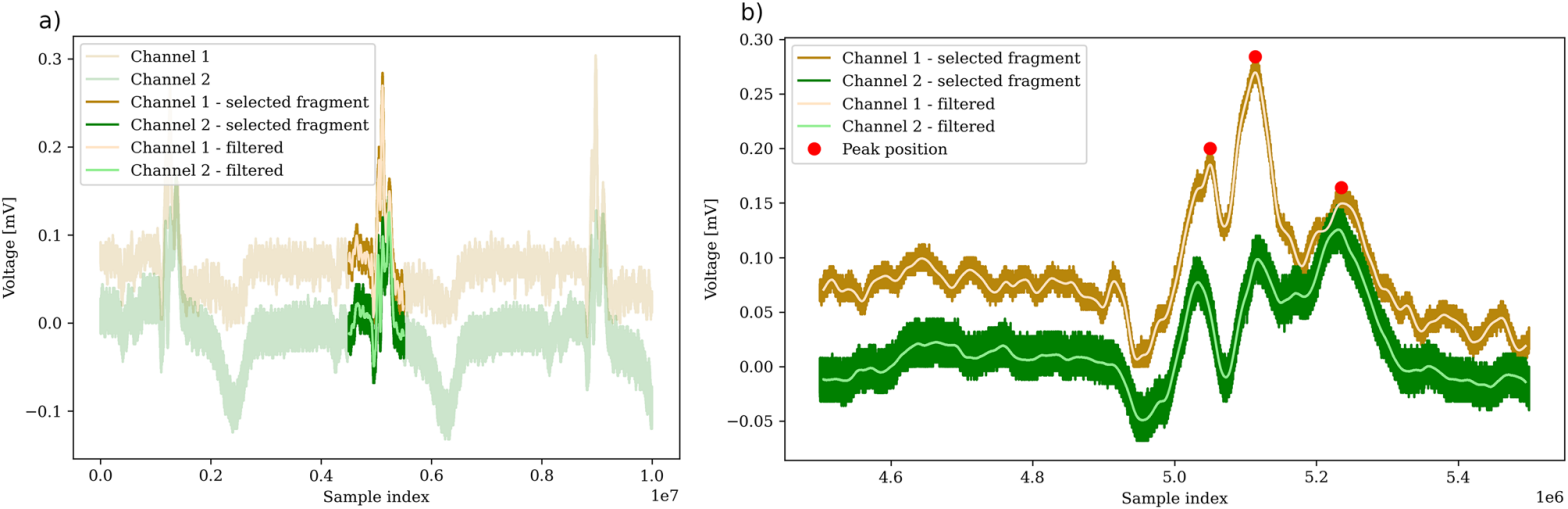
Example of the signal obtained for Subject #3. Figure (**a**) presents the wider scope of three cycles of ECG signal, Figure (**b**) presents the enlarged view of the particular QRS complex. The differences in signal morphology can be observed between Subject #1 (Fig. 2b) and Subject #3, mainly in the QRS area - for Subject #1, Q wave amplitude (downward deflection) is much bigger than for Subject #3. For Subject #3, additional low-frequency noise was discovered mainly within the QRS area. For subject #1 (the youngest participant), this noise had the smallest amplitude and did impact the uncertainty to a lesser extent. The potential causes of this noise are described in the Discussion section.

According to the Methodology protocol, first stage of measurement is to obtain ECG signals. We obtained 127 signals, which representative examples are shown in Figs. 2a and 3a for subjects #1 and #3, respectively. Figures 2b and 3b show the enlarged view of the QRS complexes for the same subjects. The recording for subject #2 was similar to #3, with QRS less distorted (detailed discussion below).

**Figure 4:**
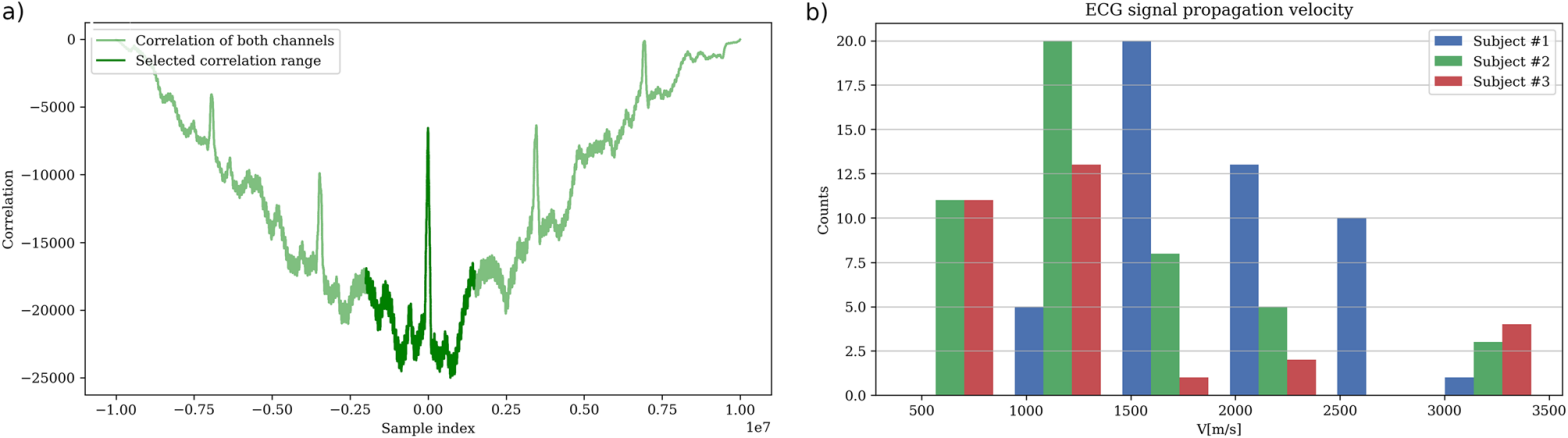
Results of ECG signal propagation velocity analysis.

Signal processing procedures performed on the signal, as described above, led to the correlation plots similar to the one shown in Fig. 4a. The analysis of the number of lag samples $\Delta s$ and application of equation 4 to the results gave values summarized in the histogram in Fig. 4b and in Table 2.

The mean value of all 150 measurements is $v=$1500(700) m/s. The median is lower: $v=$1400 m/s, as the distribution seen in Fig. 4b is skewed. The details of calculations are shown in Table 2.

**Table 2:** Results of propagation velocity measurements.

Subject	Number of samples	Mean propagation velocity [m/s]	Standard deviation [m/s]	Median [m/s]
#1	49	1800	600	1400
#2	47	1300	600	900
#3	31	1200	900	900
All	127	1500	700	1400

Number of samples indicates how many measurements were included to the overall result. For Subject #1 and #2 only 1 and 3 samples were rejected due to noise or motion artifacts. For Subject #3 the biggest impact of noise or motion artifact was observed—19 samples were rejected due to large distortions of the QRS morphology—as mentioned in text. Note, that mean propagation velocity and its median has the biggest value for Subject #1—youngest participant

## Discussion

The discussion below concerns three subjects: the quality of the results, the meaning of the results and the consequences of the result.

Concerning the quality of the result, it may be seen that the standard deviation of the measured velocity is relatively high. Relative uncertainty for subject #1 equals 33%, for subject #2 equals 46%, for subject #3 equals 75%. Such a big relative standard deviation is expected due to the dispersive character of the measured signal - each component frequency propagates with a different velocity through particular types of tissues, as described in the Introduction.

For subjects #2 and #3, higher uncertainty of measurement can be observed since for these patients, additional low-frequency noise was discovered, mainly within the area of QRS. As a result, cross-correlation occasionally gave outlying results, increasing the measurement’s uncertainty. For the subject #1 (the youngest participant), this noise had the smallest amplitude and impacted the uncertainty to a lesser extent. An example of such low-frequency noise within the QRS area observed for subject #3 is shown in Fig. 3b. Based on the peak positions (red markers), we found that period of the distortion is approximately $18.5\,ms$, which corresponds to power line interference ($54\,Hz$): reduced but not completely removed. Interestingly, it seems to have a multiplicative nature, as it does not appear outside of the QRS complex, however this can also be the artifact of filtering. For a novel, nonstandard electrode setup we have used, additional measurements are required to analyze this subject further. As we found that for subject #3, this phenomenon is less pronounced (c.f. Figs. 2b and 3b), and the quality of lag measurement is unquestioned good, we left this topic for further investigation. The harmonic as well as stochastic distortion, visible in the measured signal is a consequence of a particularly high sampling frequency which is rarely used with regard to the ECG signal. In a typical ECG measuring device, this noise would be not visible, due to the applied low pass filter at about 150 Hz. The uncertainty of muscle noise and motion artifacts can be limited by giving the subject more comfortable position such as lying down on the bed, instead of sitting on the chair. Muscle noise can’t be completely eliminated since muscle trembling is a natural phenomenon. Particular attention has to be paid to room temperature, which strongly affects the muscle noise. The artifacts connected to breathing activity can be reduced by updating the protocol of the synchronization between the signal measurement and subject’s breathing activity: The signal may be measured while the subject is asked to hold their breath at the exhaled state for 3 cycles of the heart. This should be short enough not to interfere with the subject’s breathing cycle and long enough to measure the signal with no breathing artifacts. Additionally, the grounding of Magnetical Shielding Cabin (MSC) can be improved. This may require an intervention in the power supply of the MSC and of the whole laboratory, where the measurements are performed. In order to lessen the uncertainty the skin under the electrode was prepared and wetted with ECG gel. No further preparations were performed. Alcohol cleaning, which is often used, may lead to additional cell denaturation, which may actually increase the impedance. In order to measure the distance between electrodes, a ruler was used, since the precision of this measurement of 5 cm is enough to compute the propagation velocity and confirm the original thesis, that propagation velocity is less than 30,000 m/s. For the future research on this topic more precise measurement of the distance between electrodes is recommended, in order to account for the inter-subject variability of the propagation velocity which may vary depending on age, fat percentage of the body and body hydration.

All measured values were smaller than the value *v*=30,000 m/s predicted by Eq. (3).

Many researchers have been motivated by the fact that the basic nature of tissue is its ability to conduct electric currents, and the scientific community mainly focused on conductance theories. From this point of view, dielectric permittivity does not seem a property of choice, when a physical model of propagation is proposed. This problem definitely deserves further debate. The presented work is an initial statement in this future discussion. Here we show two main theoretical results. 1) The ECG signal may be considered as an endogenous EM wave and can be modelled as such. 2) It is shown that the impedance spectrum of the tissue enables to calculate signal velocity and, generally, has an important role in the propagation of ECG signal. This approach seemed so far to be quite distant from the classical description of cardiac biopotentials.

The validation of this theoretical approach, which is supported by the results of our basic measurement, is our main experimental result. The collected evidence does not undermine existing theories: it only shows that an alternative description is viable. It should be noted that modelling of the passive propagation has been based on the Laplace equation, which was evaluated separately at each time instant. The passage of biopotential through the tissue was considered momentary. Our results show that this time is short but not infinitely short. It is interesting if any wave-related phenomena such as dispersion, reflection, diffraction or interference, for the waves of cardiac origin, can indeed be found in a living body. Of course, we have demonstrated only the electrical component of the EM wave. We can only expect that the magnetic component will be related to the electric, as predicted by Maxwell equations, but a further elaboration on this subject is out of the intended scope of this paper

Concerning the consequences of the result we obtained, they are particularly interesting for the modelling society. It has been found that the model based on the assumption of the dielectric character of the tissue will give results that are analytically indistinguishable from those obtained using volume conductor theory^18^. This discussion, fueled by current results, will be set forth in order to identify all the possible cofactors that may affect such measurements as the ECG, and to define a clear relation between the transmittance of the whole transmission line, i.e. the tissue between the heart and the location of measurement electrodes and the morphology of the ECG. Such an analysis has been limited mostly to skin preparation considerations or studies on quality of electrodes: see e.g.^16^ for a review, c.f. also^57^.

The idea that the coupling at epicardium is definitely capacitive may have further consequences for the inverse problem-solving techniques; such an analysis is currently under development. We also want to address the momentary character of tissue response, which has been assumed so far. We show, that the response of the tissue to the applied disturbance is delayed in time, which causes the finite velocity-an effect well known from solid state optics. At *f*<2 Hz, the signal is slow, as a result of slow mechanisms for tissue polarization, that are engaged. This type of behavior is shared between the tissue and all other dielectric materials^58^. We may conclude, that the quasistatic assumption used in biopotential modelling has to be revisited. It is quite probable, that there exist such mechanisms of tissue polarization which do not have enough time to develop when the state of the source changes. Note, that conductance in Purkinje fibers is 4 m/s, which is only 375 times smaller than conductance in the tissue instead of the common assumption of being infinitely slower. It is quite possible, that the slow relaxation process in the tissue may have the order of magnitude of cardiac changes: could it be the cause of mysterious U-wave^24,59^? What could be the biopotential velocity for a dehydrated patient, with an altered tissue impedance? Note, that for such a small research group, the age-related changes cannot be reliably discussed. Definitely, it does not seem to be the end of this discussion but a beginning.

## Conclusion

We have demonstrated, that the passage of biopotential of cardiac origin (ECG signal) may be treated as the propagation of endogenous electromagnetic (EM) wave through the human body. We show that within this approach, the velocity of the ECG signal propagation can be theoretically estimated, like for any other wave and physical medium, from the refraction index of the tissue in an appropriate frequency range. We confirm the theoretical predictions by the results of a direct measurement of the ECG signal propagation velocity. The results show, that the passage of an endogenous biopotential such as the ECG, through the living tissue, depends on the impedance of the tissue and the spectrum of the transmitted signal. This propagation alters the amplitude and the phase of the transmitted signal. This result links the ECG theory with the impedance spectroscopy. It may improve electrocardiographic imaging, not to mention the ECG, EEG and virtually all electric measurements.

## Study limitations and future scope

The theory and experiment presented in the above study can be expanded. In order to better estimate the propagation velocity of ECG signal through biological tissues, experimental data regarding the electrical permittivity of biological tissues in the presence of the external alternating electromagnetic field of frequencies equal and below 10Hz should be provided. In order to validate the theoretical estimation presented in this study, an experiment with a simplified model of the arm could be conducted: A model of the arm should be built from materials of known electrical permittivity spectrum, an external electromagnetic field of known frequency from range 5–20Hz should be applied to the model and the propagation velocity of this field should be measured with a method similar to the one described in this paper. This experiment could help to validate the results obtained in this study, providing also the model of dispersion curve in particular frequency range, possessed by the model of an arm. The experiment presented in this study can be improved by changing the position of the subject from sitting to lying on the wooden bed. This may reduce muscle noise. The measurement can be also taken while the subject is asked to keep their breath at the exhaled state for 3 cycles of heart rate, in order to minimize motion artifacts connected to breathing activity. Also the improved grounding of the Magnetical Shielding Cabin can potentially reduce noise and interference.

In order to explore the inter-subject variability of the propagation velocity which may vary depending on age, fat percentage of the body and body hydration, this experiment should be performed on more subjects. We also recommend more precise measurement of the distance between electrodes.

## Data Availability

All experimental data is available in the GitHub repository, http://github.com/teosbpl/HowFastIsTheECG, available from the corresponding author on reasonable request.

## Abbreviations

CH1: Channel 1
CH2: Channel 2
ECG: Electrocardiography
ECGI: Electrocardiographic imaging
EEG: Electroencephalography
EIT: Electrical impedance tomography
EM: Electromagnetic
MSC: Magnetical shielding cabin
PC: Personal computer
RF: Radio frequency

## Code availability

Source code is available in the GitHub repository, http://github.com/teosbpl/HowFastIsTheECG, available from the corresponding author on reasonable request.
